# Superficial and Deep Foveal Avascular Zone Area Measurement in Healthy Subjects Using Two Different Spectral Domain Optical Coherence Tomography Angiography Devices

**DOI:** 10.18502/jovr.v15i4.7791

**Published:** 2020-10-25

**Authors:** Pasha Anvari, Amin Najafi, Reza Mirshahi, Mahsa Sardarinia, Maryam Ashrafkhorasani, Pegah Kazemi, Gholamhoseyn Aghai, Abbas Habibi, Khalil Ghasemi Falavarjani

**Affiliations:** ^1^Eye Research Center, The Five Senses Institute, Rassoul Akram Hospital, Iran University of Medical Sciences, Tehran, Iran

**Keywords:** Fovea Avascular Zone, Optical Coherence Tomography Angiography, Optovue, Spectral-domain, Spectralis

## Abstract

**Purpose:**

To compare the area of the foveal avascular zone (FAZ) in the superficial and deep retinal layers using two different spectral-domain optical coherence tomography angiography (OCTA) devices.

**Methods:**

A cross-sectional comparative study was conducted to obtain macular OCTA images from healthy subjects using Optovue RTVue XR Avanti (Optovue, Inc, Fremont, CA) and Spectralis HRA+OCTA (Heidelberg Engineering, Heidelberg, Germany). Two independent trained graders measured the FAZ area using automated slab segmentation. The FAZ area in the superficial and deep retinal layers were compared.

**Results:**

Twenty-three eyes of 23 subjects were included. The graders agreement was excellent (>0.86) for all measurements. The mean FAZ area was significantly larger at the superficial retinal layer as compared to the deep retinal layer on both devices (0.31 ± 0.08 mm${}^{2}$ vs 0.26 ± 0.08 mm${}^{2}$ in Optovue and 0.55 ± 0.16 mm${}^{2}$ vs 0.36 ± 0.13 mm${}^{2}$ in Spectralis, both *P* < 0.001). The mean FAZ area was significantly greater in the superficial and deep retinal layers using Spectralis as compared to Optovue measurements (*P* < 0.001 for both comparisons).

**Conclusion:**

In contrast to previous reports, the FAZ area was larger in the superficial retina as compared to deep retinal layers using updated software versions. Measurements from different devices cannot be used interchangeably.

##  INTRODUCTION

Foveal avascular zone (FAZ) is the vessel-free central part of the macula surrounded by a continuous network of capillary plexus. FAZ integrity is vital for normal visual acuity. Some retinal pathologies including retinal vascular occlusions and diabetic retinopathy alter FAZ shape; therefore, FAZ metrics can serve as prognostic biomarkers for visual acuity.^[1,2,3,4,5]^ Prior to the advent of optical coherence tomography angiography (OCTA), the most extensively used tools for evaluation of FAZ were fluorescein angiography (FA) and indocyanine green angiography (ICGA).^[6,7,8]^ However, FA and ICGA are invasive procedures with side effects associated with intravenous administration of the dye.^[9]^ In addition, dye leakage from incompetent vessels may fade FAZ border in retinal pathologies, complicating FAZ assessment. OCTA is a non-invasive modality to evaluate retinal and choroidal vasculature that generates flow maps of retinal vasculature through discrimination of blood motion signals.^[10,11,12,13]^

Multiple OCTA devices are commercially available with distinct built-in software, algorithms, and techniques for visualization of retinal and choroidal vasculature and layer segmentation. The reliability and reproducibility of images provided by each device and the inter-device correlations are of great importance in clinical decision-making and patients' follow-up.^[14]^ Although several studies have reported the size of FAZ in OCTA images, automated segmentations of retinal slabs in older versions has remained a source of measurement error.^[15,16]^

The aim of this study was to measure and compare the FAZ area at the superficial and deep retinal layers using two different OCTA devices with the updated software versions.

##  METHODS

This cross-sectional comparative case-series was approved by the Iran University Ethics Committee (No. IR.IUMS.REC 1396.32837). Informed consent was obtained and the tenets of the Declaration of Helsinki were followed.

In total 23 eyes of 23 healthy employees of Rassoul Akram Hospital, without any medical conditions or ocular abnormalities, were enrolled between June 2018 and December 2018. All volunteers underwent a complete ophthalmic examination including best-corrected visual acuity (BCVA), slit-lamp examination, and fundus examination. Subjects with abnormal or suspicious findings during the examination were excluded. The right eye was considered as the target eye for OCTA imaging. All images were obtained on the same day, between 8 am and 11 am.

OCTA images were obtained using the Optovue RTVue XR Avanti (3 × 3 mm centered on the fovea, Software version 2017.1.0.15, Optovue, Inc., Fremont, CA) and Spectralis HRA+OCTA (15° × 10° scan pattern, Software version 6.9, 2017, Heidelberg Engineering, Heidelberg, Germany). The Optovue device visualizes retinal vasculature using a split-spectrum amplitude decorrelation algorithm (SSADA). The Spectralis is based on a probability algorithm which determines the presence of motion in each pixel by full spectrum amplitude-decorrelation.^[17]^

For Optovue, images with a quality score of <5 were excluded, and imaging was repeated until acceptable quality was achieved. The superficial capillary plexus (SCP) en face image was segmented automatically with an inner boundary set at the internal limiting membrane (ILM) and an outer boundary set 9 µm above the inner plexiform layer (IPL). The deep capillary plexus (DCP) en face image was segmented with an inner boundary 9 µm above the IPL and an outer boundary at 9 µm below the outer plexiform layer (OPL).

In the Spectralis device, only images with Q scores greater than 15 were included. The superficial vascular complex (SVC) was segmented automatically from ILM to 17 µm above the bottom border of the IPL and the deep vascular complex (DVC) was bounded from 17 µm above the lower border of the IPL to the bottom of the outer plexiform layer. OCTA images were imported into ImageJ software (public domain software, National Institutes of Health, Bethesda, Maryland, USA). The measurement scale was calibrated using the known dimensions of the image (3 × 3 mm for Optovue and 200 microns scale bar for Spectralis) and the FAZ boundary was outlined manually using freehand selection tools by two independent trained graders (PA and MS) in the superficial, deep, and the full retinal slabs.

Statistical data analysis was performed using the SPSS (version 17.0; SPSS Inc., Chicago, IL, USA). Intra-class correlation coefficient (ICC) between graders was calculated. The Shapiro–Wilk test was used to verify the normality of data. Paired *t*-test was used to compare mean values between two devices. The Bland–Altman plot was employed to assess the agreement between the two devices in measuring the FAZ area at different retinal layers. *P*-values < 0.05 were considered significant.

**Figure 1:**
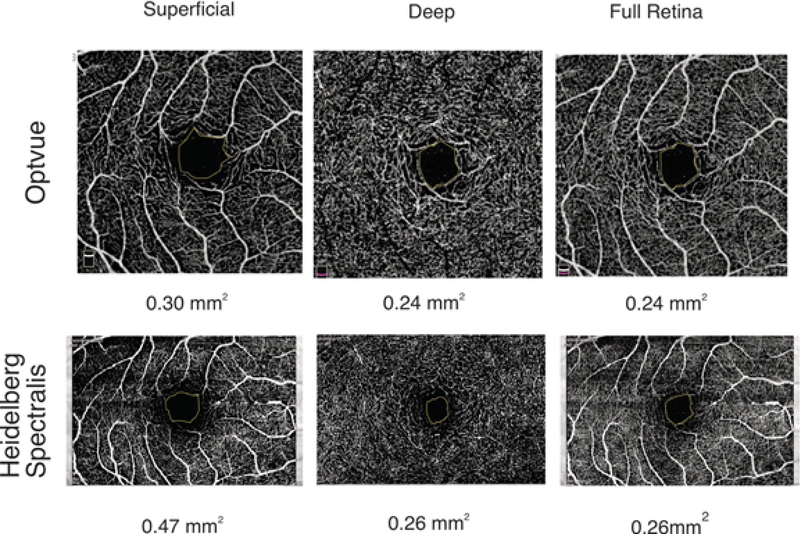
Foveal avascular zone area measurements of one eye using Optovue and Heidelberg Spectralis devices at superficial, deep, and full retinal slabs.

**Figure 2:**
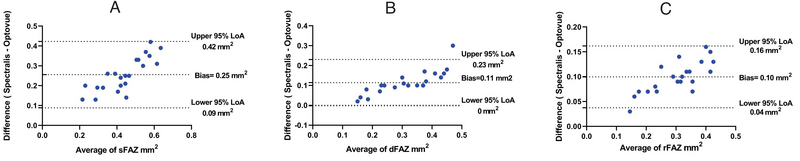
The Bland-Altman plots showing lack of agreement between Spectralis and Optovue devices in measuring FAZ area at A) superficial, B) deep and C) full retinal slabs. sFAZ, superficial foveal avascular zone; dFAZ, deep foveal avascular zone; rFAZ, foveal avascular zone in full retinal slab; LoA, Limit of Agreement

##  RESULTS

Twenty-three eyes of 23 normal subjects including 14 females (60.9%) and 9 males (39.1%) with a mean age of 34.2 ± 8.7 (range 28–59) years were included. ICC was 0.87, 0.88, and 0.86 for the measurements at superficial, deep, and full retina slabs with the Optovue device and 0.92, 0.86, and 0.88 for the measurements with the Spectralis device, respectively. In view of the excellent agreement between the graders, the mean values for the two graders were used for subsequent analysis.

Table 1 compares the FAZ measurements at the superficial and deep retinal layers with the two devices. The mean FAZ area in the superficial retinal layer was 0.55 ± 0.16 mm${}^{2}$ vs 0.31 ± 0.08 mm${}^{2}$ in the Spectralis and Optovue devices, respectively (Figure 1, *P* < 0.001). Similarly, the mean FAZ area in the deep retinal layer was significantly greater in the Spectralis as compared to the Optovue images (0.36 ± 0.13 mm${}^{2}$ vs 0.26 ± 0.08 mm${}^{2}$, *P* <0.001). The mean FAZ area of the full retina was also significantly larger with the Spectralis as compared to the Optovue device (0.36 ± 0.10 mm${}^{2}$ vs. 0.26 ± 0.07 mm${}^{2}$, *P* <0.001).

**Table 1:** Comparison of the mean foveal avascular zone area at different capillary plexuses between the Spectralis and Optovue devices


**Slab **	**Optovue**	**Spectralis**	**** ***P*** -value*
Superficial capillary plexus (mm${}^{2}$)	0.31 ± 0.08	0.55 ± 0.16	*P* < 0.001
Deep capillary plexus (mm${}^{2}$)	0.26 ± 0.08	0.36 ± 0.13	*P* < 0.001
Full retina (mm${}^{2}$)	0.26 ± 0.07	0.36 ± 0.10	*P* < 0.001
${}^{*}$Paired *t*-test			

**Table 2:** Foveal avascular zone area measured in previous studies using Optovue or Spectralis devices


**Authors**	**Superficial (mm${}^{2}$)**	**Deep (mm${}^{2}$)**	**Subjects**	**OCTA device **
Shahlaee et al, 2015^[12]^	0.27 ± 0.101	0.34 ± 0.116	17 healthy subjects	Optovue, version 2014.2.0.93
Samara et al, 2015^[19]^	0.266 ± 0.097	0.495 ± 0.227	70 eyes from 67 healthy subjects	Optovue, version 2014.2.0.13
Magrath et al, 2016^[20]^	0.2855	0.3465	50 eyes in 25 healthy volunteers	Optovue, 2014.2.0.65
Pilotto et al, 2018^[21]^	0.30 ± 0.08	0.35 ± 0.08	59 normal eyes	Spectralis unspecified version
Coscas et al, 2016^[22]^	0.28 ± 0.1	0.37 ± 0.12	135 eyes of 70 subjects	Optovue, version 2015.100.0.35
Ghassemi et al, 2017^[23]^	0.27	0.35	224 eyes of 112 volunteers	Optovue, version 2016.1.0.23-beta
Corvi et al, 2017^[17]^	Spectralis: 0.2408 Optovue: 0.2211	Spectralis: 0.3178 Optovue: 0.2619	36 eyes	Optovue, version 2016.1.0.2 Spectralis, version SP 6.7a
Mihailovic et al, 2018^[24]^	0.329	0.335	24 normal eyes	Spectralis, unspecified version
Falavarjani et al, 2018^[26]^	0.32 ± 0.11	0.50 ± 0.13	70 eyes of 70 healthy subjects	Optovue, version 2016.1.0.26
	
	

**Figure 3:**
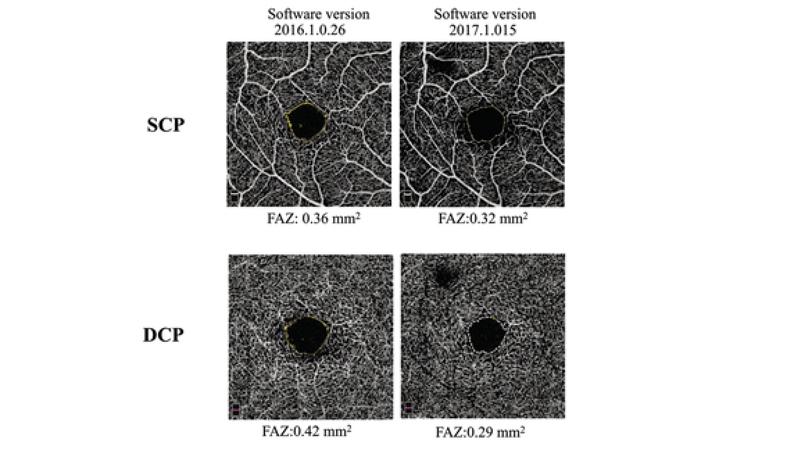
Comparison of foveal vascular zone area of one eye using two different software versions of the Optovue device. SCP, superficial capillary plexus; DCP, deep capillary plexus

With both devices, the mean FAZ area was significantly larger in the superficial retinal layer (both *P*-values < 0.001, Figure 1). None of the eyes had smaller FAZ area in the superficial retina as compared to the deep retina.

Bland–Altman plots (Figure 2) demonstrated lack of agreement between the two devices measuring the FAZ area in superficial, deep, and full retinal slabs.

##  DISCUSSION

In this study, the FAZ measurements were significantly larger in the superficial retina as compared to deep retinal layers, and both measurements were significantly greater using the Spectralis as compared to the Optovue. The OCTA provides volumetric data for different retinal layers. Each commercially available OCTA device uses a distinct built-in software for image processing and retinal layer segmentation. The details of segmentation algorithms are proprietary to manufacturers and are not publicly available. Limited studies have reported the comparisons of measurements using different devices.^[18]^ The methodology and results are conflicting between these studies.

In the current study, the FAZ area was manually determined using the ImageJ in the images captured by two spectral domain devices, Spectralis and Optovue, after automated segmentation of superficial and deep retinal layers. An excellent agreement between the independent graders was observed for both devices. Consequently, manual calculation of the FAZ area was a reliable and valid method. This is consistent with the results of previous studies reporting FAZ measurements in a single device.^[12,17,19,20,21,22,23,24,25]^

In our study, the mean FAZ area measured with Optovue was similar to previous studies (Table 2).^[12,17,19,20,21,22,23,24,25]^ However, Spectralis OCTA measurements were larger than previously reported values, particularly in the SVC. We believe that the discrepancy between our results and previous reports is a result of the recent software update and changes in segmentation reference lines. Previous versions of Spectralis segmented the SCP from the ILM to the outer boundary of IPL, and the DCP from the outer boundary of the IPL to the outer boundary of the OPL. In recent software upgrades, the slabs are segmented differently as mentioned in the Methods section. Similar to our study, Mihailovic et al^[24]^ showed that the FAZ area is larger in Spectralis images as compared to the Optovue device.

The mean FAZ area measured by Optovue was smaller than Spectralis for all measured capillary plexuses. Spaide et al^[15]^ indicated that none of the automated segmentations by three different OCTA devices (Cirrus 5000 [Carl Zeiss Meditec], RTVue XR Avanti [Optovue], and Triton DRI OCT [Topcon Medical Systems]) correlated with histological sections. Similarly, Magrath et al^[20]^ reported significant variability in FAZ measurements between Optovue and Zeiss Cirrus HD-OCT 5000 (Carl Zeiss Meditec) devices, possibly due to different slab segmentation and dissimilarities in image spatial resolution.

In contrast to previous reports, the mean FAZ area at the SCP was significantly larger than the DCP in the current study. OCTA instruments only approximately measure the SCP and DCP in lieu of the four established capillary plexuses on histological examinations,^[17]^ and current limitations of spatial resolution of different OCTA instruments may explain the observed discrepancies. Rommel et al^[16]^ revealed that the FAZ area may be different in manual segmentation of retinal layers as compared to those obtained with automated segmentations. They found that in Canon device, the mean FAZ area was greater at the SCP than DCP on manual slab segmentation simulating histologic sections.^[16]^ We used the updated versions of the Optovue and Spectralis (Figure 3), the new segmentation algorithms may be closer to histologic descriptions of retinal capillary plexuses, as described by Spaides et al.^[15]^

Our study showed the importance of taking into account the instrument type as well as the software version in assessing quantitative metrics obtained from OCTA devices. This is particularly important when assessing the progression of a disease or response to an intervention using the FAZ area on OCTA images.

We demonstrated the lack of agreement between Spectralis and Optovue in assessing FAZ area at different retinal layers. The average bias was larger when evaluating FAZ in the superficial retinal layers. Similarly, Mihailovic and colleagues^[24]^ showed slight but significant differences among Canon, Heidelberg, and Optovue in measuring the FAZ area.

One of the limitations of this study was relatively small sample size. Restricting the investigation to just two OCTA devices was another limitation. Larger studies are required to establish the relationship between FAZ measurements using different OCTA devices. In addition, the segmentation algorithm was different in the two devices. Further studies are warranted to demonstrate the repeatability of the measurements after manually changing segmentation boundaries to make them similar in different devices.

In conclusion, OCTA measurements are reliable for evaluation of FAZ area in various retinal slabs, however, commercially available devices may yield different values depending on their segmentation algorithms. Therefore, caution should be exercised in comparing measurements acquired by different OCTA devices.

##  Financial Support and Sponsorship

Nil.

##  Conflicts of Interest

There are no conflicts of interest.
